# Glial TNFα in the spinal cord regulates neuropathic pain induced by HIV gp120 application in rats

**DOI:** 10.1186/1744-8069-7-40

**Published:** 2011-05-20

**Authors:** Wenwen Zheng, Handong Ouyang, Xuexing Zheng, Shue Liu, Marina Mata, David J Fink, Shuanglin Hao

**Affiliations:** 1 Department of Neurology, University of Michigan, Ann Arbor, MI 48109, USA; 2 Department of Animal Biotechnology, College of Animal Science and Veterinary Medicine, Jilin University, Changchun 130062, Jilin Province, P. R. China; 3 Department of Anesthesiology, University of Miami Miller School of Medicine, Miami, FL33136, USA; 4 Department of Anesthesiology, State Key Laboratory of Oncology in South China, Sun Yat-Sen University Cancer Center, 651 Dongfeng Road East, Guangzhou 510060, PR China

**Keywords:** HIV pain, TNFα, glia

## Abstract

**Background:**

HIV-associated sensory neuropathy (HIV-SN) is one of the most common forms of peripheral neuropathy, affecting about 30% of people with acquired immune deficiency syndrome (AIDS). The symptoms of HIV-SN are dominated by neuropathic pain. Glia activation in the spinal cord has become an attractive target for attenuating chronic pain. This study will investigate the role of spinal TNFα released from glia in HIV-related neuropathic pain.

**Results:**

Peripheral gp120 application into the rat sciatic nerve induced mechanical allodynia for more than 7 weeks, and upregulated the expression of spinal TNFα in the mRNA and the protein levels at 2 weeks after gp120 application. Spinal TNFα was colocalized with GFAP (a marker of astrocytes) and Iba1 (a marker of microglia) in immunostaining, suggesting that glia produce TNFα in the spinal cord in this model. Peripheral gp120 application also increased TNFα in the L4/5 DRG. Furthermore, intrathecal administration of TNFα siRNA or soluble TNF receptor reduced gp120 application-induced mechanical allodynia.

**Conclusions:**

Our results indicate that TNFα in the spinal cord and the DRG are involved in neuropathic pain, following the peripheral HIV gp120 application, and that blockade of the glial product TNFα reverses neuropathic pain induced by HIV gp120 application.

## Background

Infection of the central nervous system with the human immunodeficiency virus type 1 (HIV-1) can lead to cognitive, motor and sensory disorders. HIV-associated sensory neuropathy (HIV-SN) is one of the most common forms of peripheral neuropathy, affecting about 30% of people with acquired immune deficiency syndrome (AIDS) [1,2]. The symptoms of HIV-SN are dominated by neuropathic pain [3,4]. The mechanisms underlying HIV-SN remain unclear. Astrocytosis and subsequent neuron death are two hallmarks of HIV infection in the central nervous system[5]. Direct infection of neurons by HIV is thought to be unlikely [6,7]; HIV-1 binds via the external envelope proteins (e.g., gp120) to the chemokine receptors CXCR4 and/or CCR5 (co-receptors of gp120) on the cells. Previous reports have suggested that gp120 application contributes to neurotoxicity in *in vitro *and nociceptive behaviour in rodents [8-11]. Indeed, it has been demonstrated that gp120 application is capable of producing pain when administered peripherally [12] or centrally [13]. Proposed mechanisms underlying gp120 application induced a chronic nociceptive effect included spinal gliosis [8]. HIV gp120 application might produce such effects indirectly, via an action on glial cells, causing them to release inflammatory cytokines[13].

Dysregulation of cytokines has been implicated in a variety of painful neurological diseases and in animal models of neuropathic pain. HIV-1 transgenic rats overexpressing gp120 induce reactive gliosis (in the brain), a marker for central nervous system damage [14]. HIV virus infection is able to increase the production and utilization of several cytokines, such as TNFα and IL-1β [15]. Cerebrospinal fluid from most of the patients with AIDS has increased levels of TNFα[16]. A transgenic rat developed using an HIV-1 construct, with deleted gag and pol genes, shows a strikingly high expression of TNFα [17]. A mouse model of systemic HIV-1 infection increases expression of IL-1β [18]. The viral gp120 induces the release of TNFα and IL-1β whose interaction have synergistic activities [19]; TNFα and IL-1β upregulate HIV-1 expression in cells infected by HIV [20]. This may result in an HIV gp120-cytokines reciprocal amplification with potential deleterious effects (a positive feedback cycles) [19]. An elevated baseline of TNFα level among HIV-1 positive individuals, may lead to additional neurodegeneration [21]. However, the role of spinal cytokines in the neuropathic pain induced by gp120 is not clear. In the present study, we investigated the role of TNFα in the neuropathic pain induced by gp120 application into the sciatic nerve.

## Results

### Peripheral gp120 application induced mechanical allodynia

**Figure 1:**
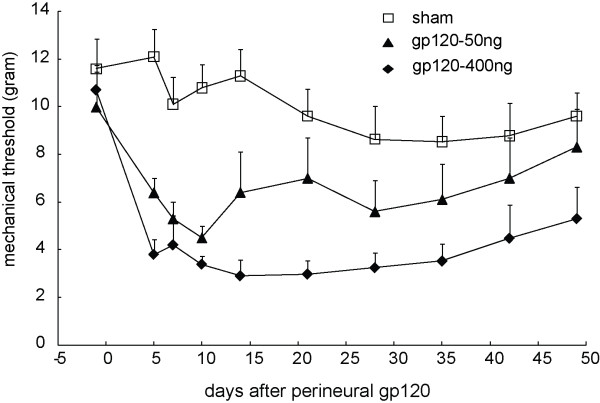
**The time course of the mechanical threshold in the model of peripheral gp120 application**. Rats exposed to perineural HIV-1 gp120 application (50 ng) developed a persistent, mechanical allodynia of the ipsilateral hind paw compared to the sham group, *F*_(1,13) _= 6.899, *p *< 0.021 vs sham, n = 7-8; HIV-1 gp120 application (400 ng) further induced mechanical allodynia of the ipsilateral hind paw, *F*_(1,14) _= 22.672, *p *< 0.001 vs sham, n = 7-9, General linear model, repeated measured, SPSS.

Before peripheral gp120 application (50 or 400 ng of gp120 in 250 μl of 0.1% RSA) into the sciatic nerve, the baseline of mechanical threshold was around 11 grams. After the gp120 application, mechanical threshold decreased significantly on the ipsilateral paw from day 5; mechanical threshold reached the lowest values at 2 weeks after gp120 application. In the sham group treated with RSA, rats showed no significant changes in mechanical threshold throughout the 7-week testing period. The difference in the threshold was very significant between the sham and gp120 application (50 ng), *F*_(1,13) _= 6.899, *p *< 0.02 vs sham, n = 7-8; high concentration of gp120 (400 ng) furthermore lowered the threshold, *F*_(1,14) _= 22.672, *p *< 0.001 vs sham, n = 7-9 (Figure 1). There is a significant difference between two doses of gp120, *F*_(1,15) _= 5.479, *p *< 0.05, n = 8, General linear model, repeated measured, SPSS (Figure 1).

### Peripheral gp120 application upregulated the expression of TNFα mRNA in the spinal cord

**Figure 2:**
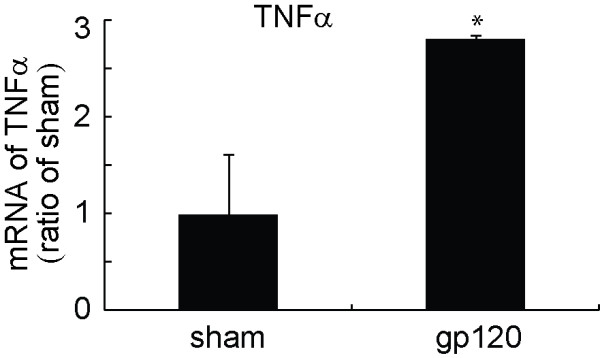
**The expression of spinal TNFα mRNA in the perineural gp120 application model using quantitative real time RT-PCR**. Two weeks after gp120 application, left L4/5 spinal dorsal horns were harvested under anesthesia and mRNA expression was examined using quantitative real time RT-PCR. Perineural gp120 application significantly upregulated spinal mRNA of TNFα. * p < 0.05 vs sham, n = 3-5, *t *test.

Original studies show that a gp120 application into the sciatic nerve induced overexpression of TNFα in the sciatic nerve[8]. In the current study, two weeks after gp120 application, the lumbar spinal dorsal horn was harvested and mRNA expression of TNFα was tested using quantitative real time RT-PCR. Application of gp120 into the nerve significantly induced the upregulation of mRNA of TNFα (Figure 2).

### Peripheral gp120 application upregulated the expression of GFAP, Iba1 and TNFα

**Figure 3:**
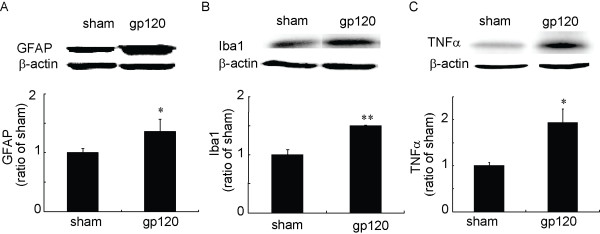
**The expression of spinal GFAP, Iba1, and TNFα in the gp120 application model**. Two weeks after gp120 application, the left L4/5 spinal dorsal horns were harvested under anesthesia, and protein expression of spinal GFAP, Iba1, and TNFα was tested using Western blots. Perineural gp120 application significantly induced the upregulation of GFAP, Iba1, and TNFα in the spinal cord level. *p < 0.05 vs sham, ** *p *< 0.01 vs sham, *t *test, n = 3-4.

Previous studies show that a gp120 application into the sciatic nerve induced overexpression of spinal GFAP (a marker of astrocytes) and OX-42 (a marker of microglia) immunoreactivity, using immunoreactive density at the spinal dorsal horn at 5, 22 and 30 days after gp120 application [8,22]. In our behavioral study (Figure 1), we observed that allodynia reached the lowest value at 2 weeks, so in the current study we focused on a 2-week timepoint to investigate the neurochemical changes. We found that the gp120 application significantly increased expression of GFAP, Iba1 (a marker of microglia), and TNFα protein in the spinal dorsal horn, compared to the sham group using Western blots (Figure 3). To our knowledge, we were the first to observe that the gp120 application increased expression of TNFα in the spinal dorsal horn.

### Examination of expression of TNFα in the spinal dorsal horn using immunohistochemistry in gp120 application-induced neuropathy

**Figure 4:**
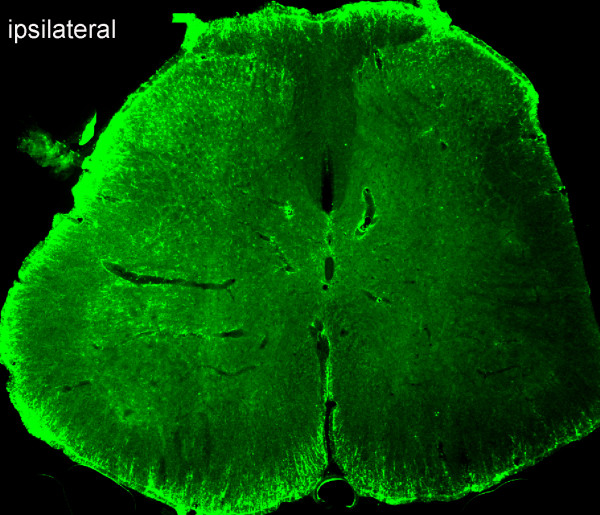
**Immunohistochemistry shows TNFα immunostaining in the whole spinal cord in rats with application of gp120 into the sciatic nerve**. Fourteen days after gp120 application, a substantial increase in the intensity of TNFα immunostaining was observed in the ipsilateral L4/5 spinal cord. The most prominent increase was found in the medial laminas I-IV of the dorsal horn, but the deep dorsal horn (laminas V and VI) and the ventral horn also showed an increase in TNFα immunostaining.

**Figure 5:**
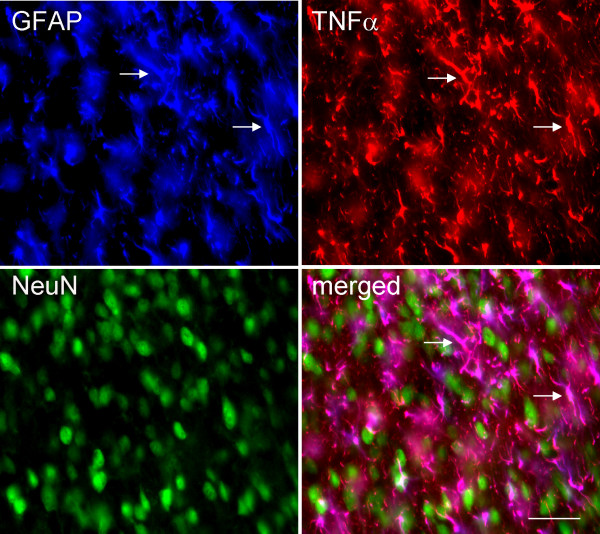
**Immunofluorescent photomicrographs of TNFα in the spinal dorsal horn in rats treated with gp120**. Triple-label immunostaining of GFAP, TNFα and NeuN was carried out. There was an almost complete colocalization between GFAP (blue) and TNFα (red) imaging, but TNFα did not colocalize with NeuN (green), which suggests that TNFα is located in the astrocytes, but not neurons. Arrow shows the colocalization.

**Figure 6:**
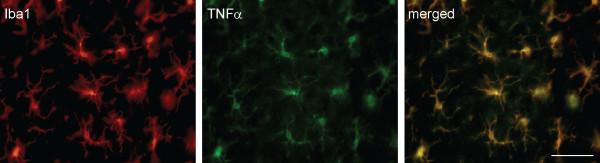
**Colocalization of TNFα and Iba1 in the spinal cord**. Double-label immunostaining of Iba1 and TNFα in the spinal dorsal horn was carried out in rats treated with gp120 application for 2 weeks. There was marked colocalization between Iba1 and TNFα imaging. Scale bar, 50 μm.

Spinal gliosis occurs following perineural HIV-gp120 application [8,11]. A substantial increase in TNFα immunoreactive staining was observed in the ipsilateral L5 spinal cord. The most prominent increase was found in the medial laminas I-IV of the dorsal horn, but the deep dorsal horn (laminas V and VI) and the ventral horn also showed an increase in TNFα immunostaining (Figure 4). However, less immunostaining of spinal TNFα was seen in the contralateral side of the gp120 application. Triple-label immunostaining of GFAP, TNFα and NeuN was carried out. There was an almost complete colocalization between GFAP (blue) and TNFα (red) imaging, but TNFα did not colocalize with NeuN (green), which suggested that TNFα was located in astrocytes, but not neurons (Figure 5). Double-label immunostaining of Iba1 and TNFα in the spinal dorsal horn was carried out in rats treated with gp120 application for 2 weeks (Figure 6). There was marked colocalization between Iba1 and TNFα imaging, suggesting that TNFα may also be released from microglia.

### Peripheral gp120 application increased the expression of TNFα in the DRG

Sensory DRG neurons play an important role in the regulation of nociceptive input transduction.

**Figure 7:**
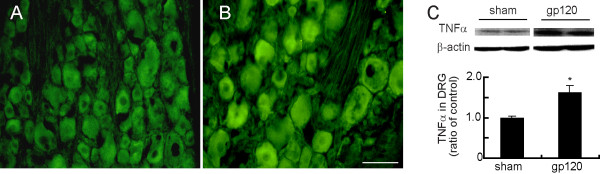
**Peripheral gp120 application increased the expression of TNFα in the DRG**. At 2 weeks after gp120 application, a clear immunostaining of TNFα in the DRG was shown (B), but TNFα in the sham group was weak (A). Bar graph showed TNFα expression in the DRG using Western blots (C), *p *< 0.05 vs sham, n = 3, *t *test.

Previous studies show that nerve injury induces upregulation of TNFα in the DRG neurons [23,24]. In the study with cultured DRG cells, CXCR4 binding on Schwann cells by gp120 application results in the release of RANTES, which induces TNFα production by DRG neurons, and subsequent TNFR1-mediated neurotoxicity in an autocrine/paracrine fashion [9]. In the current study, we observed a clear TNFα immunostaining in the DRG in rats with gp120 application (Figure 7B) compared to the sham group (Figure 7A). We found that TNFα was significantly increased in the gp120 application compared to the sham group using Western blots (Figure 7C).

#### The antinociceptive effect of glial inhibitor on the gp120 application-induced mechanical allodynia

**Figure 8:**
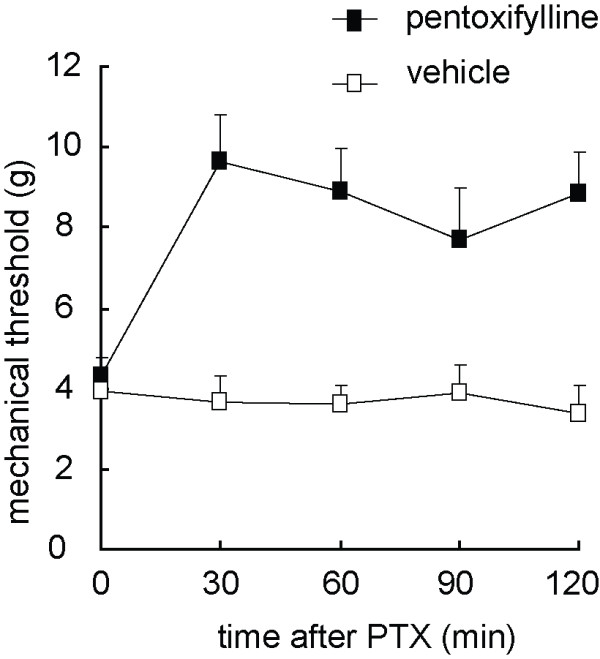
**The antinociceptive effects of glial inhibitor in rats treated with gp120 application**. At 2 weeks after gp120 application, intrathecal administration of pentoxifylline significantly reversed the allodynia in the model, *F*_(1, 8) _= 13.650, *p *= 0.006, n = 5, General linear model, repeated measure, SPSS (Figure 8).

Furthermore, we investigated the antinociceptive effect of glial inhibitor on the gp120 application-induced mechanical allodynia. Intrathecal administration of pentoxifylline, a non-specific glial cytokine inhibitor [25,26] significantly reversed the mechanical allodynia in the model (Figure 8), *F*_(1,8) _= 13.650, *p *= 0.006, n = 5, General Linear Model, repeated measure, SPSS, which was consistent with that reported in other neuropathic pain models[25,27].

### The antinociceptive effect of soluble TNF receptor on the gp120 application-induced mechanical allodynia

**Figure 9:**
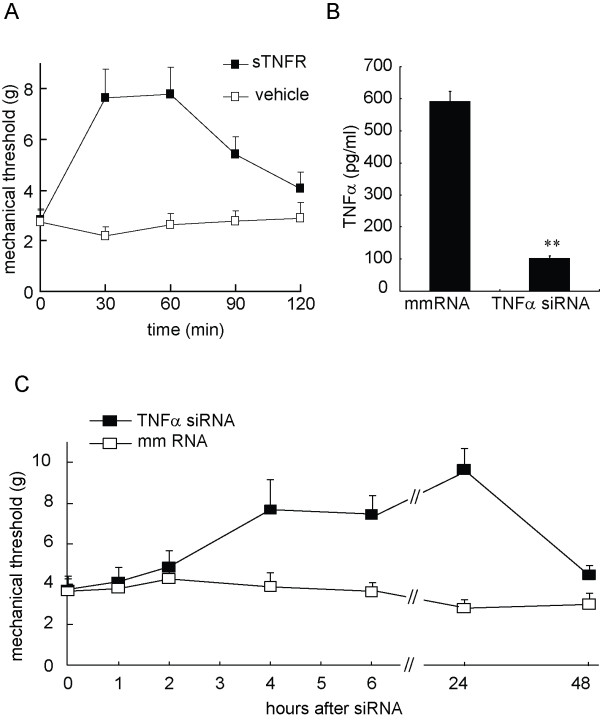
**The antinociceptive effects of recombinant soluble TNF receptor or TNFα knockdown with TNFα siRNA in rats treated with gp120 application**. (A) Rats received an intrathecal injection of recombinant soluble TNF receptor I or vehicle. Administration of recombinant soluble TNF receptor I, but not vehicle, reversed the mechanical threshold, *F*_(1, 11) _= 10.808, *p *= 0.007, n = 6-7, General linear model, repeated measure, SPSS. (B) The downregulation of TNFα in the cultured glial cell line by TNFα siRNA. The glial cells were pretreated with the TNFα siRNA or the mismatch RNA, and then stimulated with LPS. TNFα siRNA significantly lowered the TNFα expression in the cells treated with LPS, *p < 0.01, t test, n = 3. (C) The effect of intrathecal TNFα siRNA on the mechanical threshold in rats treated with gp120 application. Administration of TNFα siRNA, but not the mismatch RNA, reversed the mechanical threshold, *F*_(1, 12) _= 20.293, *p *= 0.001, n = 7, General linear model, repeated measure, SPSS.

The results above suggest that spinal TNFα plays a role in the gp120 application-induced sensory neuropathy. Furthermore, we investigated the antinociceptive effect of soluble TNF receptor on the gp120 application-induced mechanical allodynia. Soluble TNF receptor may block TNFα from binding to a membrane TNF receptor on the cell surface, to neutralize the biological effect of TNFα. Overexpression of soluble TNF receptor by HSV vectors reversed the increase in TNFα and mechanical allodynia in the neuropathic pain models [28,29]. At 2 weeks after gp120 application, intrathecal recombinant soluble TNF receptor or vehicle was administered 3 times at 12 hour intervals. Mechanical threshold was tested after the last injection. Mechanical threshold increased significantly at 30 and 60 min in rats with soluble TNF receptor, but not the vehicle (Figure 9A), *F*_(1, 11) _= 10.808, *p *= 0.007, n = 6-7, General linear model, repeated measure, SPSS.

### TNFα siRNA knockdown in vitro and in vivo

We examined whether TNFα siRNA knockdown reversed the neuropathic pain induced by gp120 application. We used cultured glial cells to identify the efficacy of TNFα siRNA that we ordered. We have found that the LPS effectively stimulates HAPI cells (one of glial cell lines) to release TNFα[30]. HAPI cells were pretreated with TNFα siRNA or mismatch RNA for 24 hours, and then stimulated with LPS for 6 hours. The supernatant was collected for testing the expression of TNFα with an ELISA kit (R&D). Pretreatment with TNFα siRNA, but not mismatch RNA suppressed the expression of TNFα in the cultured medium (Figure 9B). In the control group without LPS, non TNFα was detected (data not shown). Intrathecal siRNA of TNFα or mismatch RNA (10 μg) was given 2 times at 12-hour intervals. Mechanical threshold was tested after the last injection, and increased significantly at 6 and 24 hours in rats with TNFα siRNA, but not the mismatch RNA (Figure 9C), *F*_(1,12) _= 20.293, *p *= 0.001, n = 7, General Linear Model, repeated measure with SPSS.

## Discussion

Previous studies indicate that TNFα is involved in the development of chronic pain. There is growing evidence suggesting that glial activation plays an important role in the HIV-sensory neuropathy. The current study showed 1) that HIV gp120 application into the sciatic nerve induced neuropathic pain behavior, and upregulated the expression of spinal GFAP, Iba1, and TNFα; 2) that TNFα was colocalized with either GFAP or Iba1 in the spinal cord, suggesting that TNFα is released from the activated astrocytes or microglia; 3) that gp120 application also induced upregulation of TNFα in the DRG; and 4) that knockdown of TNFα with siRNA or recombinant soluble TNF receptor reversed mechanical allodynia induced by gp120 application.

Neuropathic pain is disorder resulting from damage or alteration to nerve structures in the absence of demonstrated tissue damage. HIV infection might influence the basic neurobiology, neurological morphology, and clinical management of neurological dysfunction [31-33]. The entry of HIV into cells requires the sequential interaction of the viral exterior envelope glycoprotein, gp120 (cleavage of gp160), with the CD4 glycoprotein and chemokine receptors on the cell surface [34-37], facilitating receptor signaling in both the peripheral nervous system and the CNS [36,38,39]. In *in vitro *studies, HIV-gp120 binding to Schwann cells through CXCR4 results in the release of RANTES, which induces TNFα production by DRG, and subsequent TNFR1-mediated neurotoxicity in an autocrine/paracrine fashion [9]. In *in vivo *studies, HIV-1 transgenic rats overexpressing gp120 induce reactive gliosis in brain [14]. Astrocyte activation or astrocytosis may directly contribute to HIV-associated neurological disorders [40]. Injection of gp120 into the hindpaws produces pain hypersensitivity by directly exciting primary nociceptive neurons [41]. Intrathecal injection of gp120 recombinant protein induces an acute painful behavior and proinflammatory cytokine release in the spinal cord [10]. Cerebrospinal fluid from most patients with AIDS shows an increase in TNFα [16]. The HIV gp120 induces the release of IL-1β and TNFα whose interaction has synergistic activities [19]. In clinic, TNFα has also been implicated in the pathogenesis of HIV-1 infection, promoting HIV replication in T cell lines and in lymphocytes in HIV-infected patients [42]. Serum concentrations of TNFα have been shown to increase as HIV-1 infection progresses[43], suggesting that TNFα may contribute to disease progression. Thus, inhibition of TNFα in the setting of HIV infection has been appealing, at least in theory. However, whether TNFα is involved in the development of neuropathic pain in the HIV/AIDS patients is not clear.

Inflammation of peripheral nerves causes sustained increased electrical activity in the C/Aδ fibers, that leads to transcriptional and post-translational changes in second order neurons in the spinal dorsal horn, that are characteristic of chronic pain [44]. Evidence indicates that peripheral nerve damage or inflammation, results in the activation of glia in the dorsal horn that plays an important role in the pathogenesis of neuropathic pain [45-47]. After peripheral nerve injury or spinal cord injury TNFα in spinal microglia or astrocytes is increased [28,29,46]. In the current study, we used the peripheral gp120 application model and also found similar results.

In the chronic constriction injury model of peripheral neuropathic pain, neutralizing antibodies to TNF and to TNFR1 reduce thermal hyperalgesia and mechanical allodynia[48], and intrathecal administration of the recombinant soluble TNFR (sTNFR) peptide (etanercept), prior to selective spinal nerve ligation reduces mechanical allodynia [40]. Administration of drugs that block the effects of these cytokines [24,49] or that block glial activation [50] can be used to prevent or reverse neuropathic pain, which is consistent with our results. Previous studies have shown that overexpression of spinal TNFα released from microglia and/or astrocytes play an important role in the different neuropathic pain models [28,29,51,52]. Our current study showed that TNFα in the DRG might also involve neuropathic pain in this model, which is consistent with previous reports [23,24].

Transmembrane TNFα, a precursor of the soluble form of TNFα (sTNFα), is expressed on activated macrophages and lymphocytes as well as other cell types (e.g. glia in the CNS). After processed by TNF-alpha-converting enzyme, the soluble form of TNFα is cleaved from transmembrane TNFα and mediates its biological activities[53]. Although many studies demonstrate increased TNFα mRNA and/or protein in neuropathic pain, to our knowledge, none of those reports demonstrate the release of sTNFα in the spinal cord in models of persistent pain. In our previous studies of neuropathic pain induced by spinal cord injury [28], spinal nerve ligation[29], and of inflammatory pain [30], we have found by Western blot that there is an increase in full-length mTNFα (26 kD) without detectable sTNFα in the spinal dorsal horn. In the current study, we did not observe sTNFα either.

In summary, there is abundant evidence to suggest that one of the important elements is neuroimmune activation of glia and glial products in the spinal cord in the neuropathic pain state [54-56]. While the mechanisms underlying HIV-related neuropathic pain are poorly understood, the results of the current investigation provide an important insight into the pathogenesis of chronic pain. Other targets (e.g., IL-1β, p-p38) will be addressed in the near future.

## Methods

### Animal experiments

Male Sprague-Dawley rats weighing 225-250 g were housed one to three per cage approximately 7 days prior to the beginning of the study. Free access to food and water and maintained on a 12:12, light: dark schedule at 21°C and 60% humidity. All housing conditions and experimental procedures were approved by the University Animal Care and Use Committee and were conducted in accordance with the ethical guidelines of the International Association for the Study of Pain[57].

### Intrathecal catheter implantation

For intrathecal administration, intrathecal catheters were implanted under isoflurane anesthesia[58]. A polyethylene (PE-10) catheter filled with 0.9% saline was advanced 8 cm caudally through an incision in the atlanto-occipital membrane to position its tip at the level of the lumbar enlargement. The rostral tip of the catheter was passed subcutaneously, externalized on top of the skull, and sealed with a stainless-steel plug. Animals showing neurological deficits after implantation were excluded. Animals were used within 5 days after implantation of the catheter.

#### Perineural gp120 application model

Under anesthesia, male Sprague-Dawley rats (225-250 grams) were used and the left sciatic nerve was exposed in the popliteal fossa without damaging the nerve construction. A 2 × 6 mm strip of oxidized regenerated cellulose was previously soaked in 250 μl of a 0.1% rat serum albumin (RSA) in saline, containing 50 or 400 ng of gp120 (Immunodiagnostics, Bedford, MA.) or 0.1% RSA in saline for the sham surgery. A length 3-4 mm of sciatic nerve was wrapped loosely with the previously soaked cellulose, proximal to the trifurcation not to cause any nerve constriction and left *in situ *[8,22]. The incision was closed with 4/0 sutures. Application of gp120 induced a marked decrease in the mechanical threshold.

### Mechanical threshold

Animals were placed in transparent plastic cubicles on a mesh floor for an acclimatization period of at least 30 min on the morning of the test day. Mechanical allodynia was determined by assessing paw withdrawal to von Frey hairs of graded tensile strength. A series of calibrated von Frey filaments were presented serially to the hind paw in ascending order of strength, with each filament applied for 6 s with sufficient force to cause slight bending against the paw. A positive response was defined as rapid withdrawal and/or licking of the paw immediately upon application of the stimulus, which was then followed by application of the next finer von Frey filament. After a negative response, the next higher von Frey filament was applied. Animals that did not respond to a pressure of 15.1 g were assigned to this cutoff value. The tactile stimulus producing a 50% likelihood of withdrawal was determined using the up-down method [59,60]

#### Quantitative real-time polymerase chain reaction (PCR)

Total RNA was isolated from the spinal cord using TRIzol reagent (Invitrogen, Camarillo, CA, USA), treated with RNase-free DNase-I (Roche, Indianapolis, IN, USA) and re-purified, and then quantified spectrophotometrically. Total RNA (1 μg) was reverse transcribed (Omniscript RT kit, Qiagen, Valencia, CA, USA) using random hexamers PCR primer. cDNA prepared from mRNA was amplified using the following primer sets: GAPDH-forward 5'-GTTTGTGATGGGTGTGAACC-3' and -reverse 5'-TCTTCTGAGTGGCAGTGATG-3'; TNFα-forward 5'-CTTCAAGGGACAAGGCTG-3' and -reverse 5'-GAGGCTGACTTTCTCCTG-3'. PCR was performed with equal amounts of cDNA in the GeneAmp 7700 sequence detection system (Applied Biosystems, Foster City, CA, USA), using SYBR^® ^Green PCR Master Mix (Applied Biosystems, Foster City, CA, USA). Reactions (total volume, 25 μl) were incubated at 95°C for 10 min, followed by 40 cycles of 15 s at 95°C and 1 min at 60°C. Each sample was measured, and data points were examined for integrity by analysis of the amplification plot. The comparative cycle threshold (Ct) method was used for relative quantification of gene expression. The amount of mRNA, normalized to the endogenous control (GAPDH) and relative to a calibrator, is given by 2^-^ΔΔ^Ct^, with Ct indicating the cycle number at which the fluorescence signal of the PCR product crosses an arbitrary threshold set within the exponential phase of the PCR, and ΔΔCt = [(Ct_target (unknown sample) _- Ct_end.control __(unknown sample)_)] - [(Ct_target (calibrator sample) _- Ct_end. control (calibrator sample)_)] as previously described [61].

#### Western Blots

The tissues were homogenized in protein lysis buffer (150 mM sodium chloride, 1.0% NP-40, 0.5% sodium deoxycholate, 0.1% SDS, 50 mM Tris, pH 8.0) containing protease inhibitors and phosphatase inhibitors (Phosphatase Inhibitor Cocktails 1/2, Sigma, St. Louis, MO, USA). The homogenate was centrifuged at 18,000 g for 20 min at 4°C. The supernatant was collected and assayed for protein concentration using the DC protein assay (Bio-Rad, Hercules, CA, USA). Aliquots containing 30 μg of protein were dissolved in Laemmli buffer and denatured at 95°C for 5 min; the proteins were separated by 10% Tris-glycine SDS-PAGE gel and transferred to a PVDF membrane. The membranes were blocked with 5% nonfat dry milk in PBS buffer, and then incubated with primary antibodies for 1 h at room temperature, including mouse anti-GFAP (1:10,000, Sigma, St. Louis, MO, USA), rabbit anti-Iba1 antibody (1:1000, Wako, Richmond, VA), rabbit polyclonal anti-TNFα (1 : 500, Chemicon, Temecula, CA) and mouse anti-*β*-actin, 1 : 8000, monoclonal antibody, Sigma, St. Louis, MO, USA). The blots were incubated with secondary antibodies (Santa Cruz Biotechnology, Santa Cruz, CA, USA), developed in chemiluminescence solution (Thermo Scientific, Rockford, IL USA). Quantification of Western blots was done from the obtained chemiluminescence values (BioRad, Hercules, CA, USA). Target protein bands were normalized using the amount of β-actin.

### Immunohistochemistry

Immunohistochemical expression of GFAP, Iba1, TNFα and NeuN in the spinal cord in rats with gp120 treatment was investigated as described previously [62]. For immunofluorescence detection, cryosections were probed overnight with rabbit anti-GFAP polyclonal antibody (1 : 2000, DakoCytomation, Glostrup, Denmark), rabbit anti-Iba1 antibody (1:1000, Wako, Richmond, VA), goat anti-rat TNFα antibody (1 : 100; R&D systems, Minneapolis, MN), mouse anti-NeuN monoclonal antibody (A60) (1 : 5000, Millipore, Billerica, MA), and then followed by incubation with complementary secondary antibodies labeled with blue-fluorescent Alexa Fluor 350, green-fluorescent Alexa Fluor 488, or red-fluorescent Alexa Fluor 594 (1 : 2000, Molecular Probes, Eugene, OR), 2 h at room temperature and photographed using a fluorescence microscope. Sections were selected and scanned using a Nikon fluorescence microscope.

### Evaluation of the effect of TNFα siRNA in vitro

Glial cell line was used to verify the efficiency of TNFα siRNA *in vitro*. TNFα siRNAs (forward 5'-GCCCGUAGCCCACGUCGUAdTdT-3', reverse-5'-UACGACGUGGGCUACGGGCdTdT-3') and mismatch siRNA (forward-5'-GCCCGUAGAACACGUCGUAdTdT-3', reverse-5'-UACGACGUGUUCUACGGGCdTdT-3') were synthesized by Invitrogen (Invitrogen, CA, USA). We demonstrated that cultured HAPI cells (a glial cell line) treated with LPS, released TNFα [30]. In this study, HAPI cells were seeded into 6-well plates at 2 × 10^5 ^cells/well 24 h before transfection. Transient transfection procedures were performed according to Lipofectamine RNAiMAX reagent instructions (Invitrogen, Camarillo, CA, USA). Briefly, HAPI cells were incubated with 100 pmol siRNA and 6 μl Lipofectamine RNAiMAX complexes in Opti-MEM I reduced serum medium (Invitrogen, Camarillo, CA, USA). Twenty four hours after siRNA application, cells were stimulated by 1 ug/ml of LPS (Sigma, St. Louis, MO, USA). Six hours after LPS, the supernatant was harvested and the concentration of TNFα was measured using an ELISA kit (R&D Systems, Minneapolis, MN, USA) according to the manufacturer's instructions.

### Drugs and Data Analysis

Recombinant gp120 was purchased from Immunodiagnostics (Woburn, MA) and dissolved in 0.1% RSA. Soluble TNF receptor I was purchased from PeproTech and dissolved in 0.1% RSA. Pentoxifylline was purchased from Sigma (Sigma, St. Louis, MO) and dissolved in saline. The drug doses were selected on the basis of previous reports and our preliminary studies. To facilitate siRNA into cells, we used polyethyleneimine (PEI), a cationic polymer, as a delivery vehicle to prevent degradation and enhance cell membrane penetration of siRNA [63]. siRNA was dissolved in RNase-free water at the concentration of 1 μg/μl as a stock solution. Ten min before injection, 10 μl siRNA was mixed with 1.8 μl PEI. Intrathecal drugs were injected through the implanted polyethylene tubing (PE-10) within 10 μl followed by 10 μl of saline; the injection lasted 30 s by means of a Hamilton syringe.

The statistical significance of the differences was determined by the *t *test. The difference between the time-course curves of the behavioral testing was determined using a General Linear Model, repeated measure with SPSS. *P*-values of less than 0.05 were considered to be statistically significant.

## Competing interests

David Fink receives compensation for professional services from the University of Michigan and from the Department of Veterans Affairs. He also receives payments from the University of Pittsburgh for patents owned by the University on which he is a co-inventor. None of the other authors have received compensation for professional services or anticipate receiving such compensation in the near future.

## Authors' contributions

WZ participated in RT-PCR, and Western blot. HO performed the surgery and behavioral testing. XZ was involved in siRNA and behavioral testing with HO. SL carried out sample collection and immunohistochemistry. MM and DF participated in the data analysis and interpretation. SH contributed to the experimental designs, the data analysis and interpretation, and wrote the manuscript. All authors reviewed and approved the final manuscript.

## References

[B1] Schifitto G McDermott MP McArthur JC Marder K Sacktor N Epstein L Kieburtz K Incidence of and risk factors for HIV-associated distal sensory polyneuropathy Neurology 2002 58 1764 1768 12084874 10.1212/wnl.58.12.1764

[B2] Simpson DM Kitch D Evans SR McArthur JC Asmuth DM Cohen B Goodkin K Gerschenson M So Y Marra CM HIV neuropathy natural history cohort study: assessment measures and risk factors Neurology 2006 66 1679 1687 10.1212/01.wnl.0000218303.48113.5d 16769940

[B3] Newshan G Pain in human immunodeficiency virus disease Semin Oncol Nurs 1997 13 36 41 10.1016/S0749-2081(97)80048-4 9048435

[B4] Dorsey SG Morton PG HIV peripheral neuropathy: pathophysiology and clinical implications AACN Clin Issues 2006 17 30 36 10.1097/00044067-200601000-00004 16462406

[B5] Zhou BY Liu Y Kim B Xiao Y He JJ Astrocyte activation and dysfunction and neuron death by HIV-1 Tat expression in astrocytes Mol Cell Neurosci 2004 27 296 305 15519244 10.1016/j.mcn.2004.07.003

[B6] Michaels J Sharer LR Epstein LG Human immunodeficiency virus type 1 (HIV-1) infection of the nervous system: a review Immunodefic Rev 1988 1 71 104 3078711

[B7] Lipton SA Neuronal injury associated with HIV-1 and potential treatment with calcium-channel and NMDA antagonists Dev Neurosci 1994 16 145 151 10.1159/000112101 7705221

[B8] Herzberg U Sagen J Peripheral nerve exposure to HIV viral envelope protein gp120 induces neuropathic pain and spinal gliosis J Neuroimmunol 2001 116 29 39 10.1016/S0165-5728(01)00288-0 11311327

[B9] Keswani SC Polley M Pardo CA Griffin JW McArthur JC Hoke A Schwann cell chemokine receptors mediate HIV-1 gp120 toxicity to sensory neurons Ann Neurol 2003 54 287 296 10.1002/ana.10645 12953261

[B10] Milligan ED O'Connor KA Nguyen KT Armstrong CB Twining C Gaykema RP Holguin A Martin D Maier SF Watkins LR Intrathecal HIV-1 envelope glycoprotein gp120 induces enhanced pain states mediated by spinal cord proinflammatory cytokines J Neurosci 2001 21 2808 2819 11306633 10.1523/JNEUROSCI.21-08-02808.2001 PMC6762530

[B11] Wallace VC Blackbeard J Pheby T Segerdahl AR Davies M Hasnie F Hall S McMahon SB Rice AS Pharmacological, behavioural and mechanistic analysis of HIV-1 gp120 induced painful neuropathy Pain 2007 133 47 63 10.1016/j.pain.2007.02.015 17433546 PMC2706950

[B12] Eron JJ Jr Ashby MA Giordano MF Chernow M Reiter WM Deeks SG Lavelle JP Conant MA Yangco BG Pate PG Randomised trial of MNrgp120 HIV-1 vaccine in symptomless HIV-1 infection Lancet 1996 348 1547 1551 10.1016/S0140-6736(96)05283-X 8950881

[B13] Milligan ED Mehmert KK Hinde JL Harvey LO Martin D Tracey KJ Maier SF Watkins LR Thermal hyperalgesia and mechanical allodynia produced by intrathecal administration of the human immunodeficiency virus-1 (HIV-1) envelope glycoprotein, gp120 Brain Res 2000 861 105 116 10.1016/S0006-8993(00)02050-3 10751570

[B14] Reid W Sadowska M Denaro F Rao S Foulke J Jr Hayes N Jones O Doodnauth D Davis H Sill A An HIV-1 transgenic rat that develops HIV-related pathology and immunologic dysfunction Proc Natl Acad Sci USA 2001 98 9271 9276 10.1073/pnas.161290298 11481487 PMC55410

[B15] Merrill JE Chen IS HIV-1, macrophages, glial cells, and cytokines in AIDS nervous system disease FASEB J 1991 5 2391 2397 2065887 10.1096/fasebj.5.10.2065887

[B16] Tyor WR Glass JD Griffin JW Becker PS McArthur JC Bezman L Griffin DE Cytokine expression in the brain during the acquired immunodeficiency syndrome Ann Neurol 1992 31 349 360 10.1002/ana.410310402 1586135

[B17] Cedeno-Laurent F Bryant J Fishelevich R Jones OD Deng A Eng ML Gaspari AA Trujillo JR Inflammatory papillomatous hyperplasia and epidermal necrosis in a transgenic rat for HIV-1 J Dermatol Sci 2009 53 112 119 10.1016/j.jdermsci.2008.08.015 19004620 PMC3125122

[B18] Potash MJ Chao W Bentsman G Paris N Saini M Nitkiewicz J Belem P Sharer L Brooks AI Volsky DJ A mouse model for study of systemic HIV-1 infection, antiviral immune responses, and neuroinvasiveness Proc Natl Acad Sci USA 2005 102 3760 3765 10.1073/pnas.0500649102 15728729 PMC553332

[B19] Ilyin SE Plata-Salaman CR HIV-1 envelope glycoprotein 120 regulates brain IL-1beta system and TNF-alpha mRNAs in vivo Brain Res Bull 1997 44 67 73 10.1016/S0361-9230(97)00091-9 9288832

[B20] Yamamoto N The role of cytokines in the acquired immunodeficiency syndrome Int J Clin Lab Res 1995 25 29 34 10.1007/BF02592573 7787207

[B21] Kumar M Kumar AM Waldrop D Antoni MH Eisdorfer C HIV-1 infection and its impact on the HPA axis, cytokines, and cognition Stress 2003 6 167 172 10.1080/10253890310001605376 13129810

[B22] Wallace VC Blackbeard J Segerdahl AR Hasnie F Pheby T McMahon SB Rice AS Characterization of rodent models of HIV-gp120 and anti-retroviral-associated neuropathic pain Brain 2007 130 2688 2702 10.1093/brain/awm195 17761732 PMC2656646

[B23] He XH Zang Y Chen X Pang RP Xu JT Zhou X Wei XH Li YY Xin WJ Qin ZH Liu XG TNF-alpha contributes to up-regulation of Nav1.3 and Nav1.8 in DRG neurons following motor fiber injury Pain 2010 151 266 279 10.1016/j.pain.2010.06.005 20638792

[B24] Schafers M Svensson CI Sommer C Sorkin LS Tumor necrosis factor-alpha induces mechanical allodynia after spinal nerve ligation by activation of p38 MAPK in primary sensory neurons J Neurosci 2003 23 2517 2521 12684435 10.1523/JNEUROSCI.23-07-02517.2003 PMC6742090

[B25] Mika J Osikowicz M Rojewska E Korostynski M Wawrzczak-Bargiela A Przewlocki R Przewlocka B Differential activation of spinal microglial and astroglial cells in a mouse model of peripheral neuropathic pain Eur J Pharmacol 2009 623 65 72 10.1016/j.ejphar.2009.09.030 19766105

[B26] Shohami E Bass R Wallach D Yamin A Gallily R Inhibition of tumor necrosis factor alpha (TNFalpha) activity in rat brain is associated with cerebroprotection after closed head injury J Cereb Blood Flow Metab 1996 16 378 384 8621742 10.1097/00004647-199605000-00004

[B27] Saito O Svensson CI Buczynski MW Wegner K Hua XY Codeluppi S Schaloske RH Deems RA Dennis EA Yaksh TL Spinal glial TLR4-mediated nociception and production of prostaglandin E(2) and TNF Br J Pharmacol 2010 160 1754 1764 10.1111/j.1476-5381.2010.00811.x 20649577 PMC2936846

[B28] Peng XM Zhou ZG Glorioso JC Fink DJ Mata M Tumor necrosis factor-alpha contributes to below-level neuropathic pain after spinal cord injury Ann Neurol 2006 59 843 851 10.1002/ana.20855 16634039

[B29] Hao S Mata M Glorioso JC Fink DJ Gene transfer to interfere with TNFalpha signaling in neuropathic pain Gene Ther 2007 14 1010 1016 10.1038/sj.gt.3302950 17443214

[B30] Zhou Z Peng X Hao S Fink DJ Mata M HSV-mediated transfer of interleukin-10 reduces inflammatory pain through modulation of membrane tumor necrosis factor alpha in spinal cord microglia Gene Ther 2008 15 183 190 10.1038/sj.gt.3303054 18033311 PMC2572752

[B31] Gelman BB Spencer JA Holzer CE Soukup VM Abnormal striatal dopaminergic synapses in National NeuroAIDS Tissue Consortium subjects with HIV encephalitis J Neuroimmune Pharmacol 2006 1 410 420 10.1007/s11481-006-9030-6 18040813

[B32] Jernigan TL Gamst AC Archibald SL Fennema-Notestine C Mindt MR Marcotte TD Heaton RK Ellis RJ Grant I Effects of methamphetamine dependence and HIV infection on cerebral morphology Am J Psychiatry 2005 162 1461 1472 10.1176/appi.ajp.162.8.1461 16055767

[B33] Meeker RB Feline immunodeficiency virus neuropathogenesis: from cats to calcium J Neuroimmune Pharmacol 2007 2 154 170 10.1007/s11481-006-9045-z 18040840 PMC3166855

[B34] Roth MD Tashkin DP Whittaker KM Choi R Baldwin GC Tetrahydrocannabinol suppresses immune function and enhances HIV replication in the huPBL-SCID mouse Life Sci 2005 77 1711 1722 10.1016/j.lfs.2005.05.014 15964028

[B35] Mahajan SD Schwartz SA Nair MP Immunological assays for chemokine detection in in-vitro culture of CNS cells Biol Proced Online 2003 5 90 102 10.1251/bpo50 12734551 PMC153847

[B36] Berman JW Carson MJ Chang L Cox BM Fox HS Gonzalez RG Hanson GR Hauser KF Ho WZ Hong JS NeuroAIDS, drug abuse, and inflammation: building collaborative research activities J Neuroimmune Pharmacol 2006 1 351 399 10.1007/s11481-006-9048-9 18040811

[B37] Williams KC Burdo TH HIV and SIV infection: the role of cellular restriction and immune responses in viral replication and pathogenesis APMIS 2009 117 400 412 10.1111/j.1600-0463.2009.02450.x 19400864 PMC2739573

[B38] Kaul M Zheng J Okamoto S Gendelman HE Lipton SA HIV-1 infection and AIDS: consequences for the central nervous system Cell Death Differ 2005 12 Suppl 1 878 892 15832177 10.1038/sj.cdd.4401623

[B39] Garzino-Demo A DeVico AL Conant KE Gallo RC The role of chemokines in human immunodeficiency virus infection Immunol Rev 2000 177 79 87 10.1034/j.1600-065X.2000.17711.x 11138787

[B40] Zou W Kim BO Zhou BY Liu Y Messing A He JJ Protection against human immunodeficiency virus type 1 Tat neurotoxicity by Ginkgo biloba extract EGb 761 involving glial fibrillary acidic protein Am J Pathol 2007 171 1923 1935 10.2353/ajpath.2007.070333 18055541 PMC2111115

[B41] Oh SB Tran PB Gillard SE Hurley RW Hammond DL Miller RJ Chemokines and glycoprotein120 produce pain hypersensitivity by directly exciting primary nociceptive neurons J Neurosci 2001 21 5027 5035 11438578 10.1523/JNEUROSCI.21-14-05027.2001 PMC6762869

[B42] Cepeda EJ Williams FM Ishimori ML Weisman MH Reveille JD The use of anti-tumour necrosis factor therapy in HIV-positive individuals with rheumatic disease Ann Rheum Dis 2008 67 710 712 18079191 10.1136/ard.2007.081513

[B43] Aukrust P Liabakk NB Muller F Lien E Espevik T Froland SS Serum levels of tumor necrosis factor-alpha (TNF alpha) and soluble TNF receptors in human immunodeficiency virus type 1 infection--correlations to clinical, immunologic, and virologic parameters J Infect Dis 1994 169 420 424 10.1093/infdis/169.2.420 7906293

[B44] Scholz J Woolf CJ Can we conquer pain? Nat Neurosci 2002 5 Suppl 1062 1067 12403987 10.1038/nn942

[B45] Hashizume H DeLeo JA Colburn RW Weinstein JN Spinal glial activation and cytokine expression after lumbar root injury in the rat Spine (Phila Pa 1976) 2000 25 1206 1217 10.1097/00007632-200005150-00003 10806496

[B46] Raghavendra V Rutkowski MD DeLeo JA The role of spinal neuroimmune activation in morphine tolerance/hyperalgesia in neuropathic and sham-operated rats J Neurosci 2002 22 9980 9989 12427855 10.1523/JNEUROSCI.22-22-09980.2002 PMC6757841

[B47] Homma Y Brull SJ Zhang JM A comparison of chronic pain behavior following local application of tumor necrosis factor alpha to the normal and mechanically compressed lumbar ganglia in the rat Pain 2002 95 239 246 10.1016/S0304-3959(01)00404-3 11839423

[B48] Sommer C Schmidt C George A Hyperalgesia in experimental neuropathy is dependent on the TNF receptor 1 Exp Neurol 1998 151 138 142 10.1006/exnr.1998.6797 9582261

[B49] Sweitzer S Martin D DeLeo JA Intrathecal interleukin-1 receptor antagonist in combination with soluble tumor necrosis factor receptor exhibits an anti-allodynic action in a rat model of neuropathic pain Neuroscience 2001 103 529 539 10.1016/S0306-4522(00)00574-1 11246166

[B50] Sweitzer SM Schubert P DeLeo JA Propentofylline, a glial modulating agent, exhibits antiallodynic properties in a rat model of neuropathic pain J Pharmacol Exp Ther 2001 297 1210 1217 11356948

[B51] Xu JT Xin WJ Zang Y Wu CY Liu XG The role of tumor necrosis factor-alpha in the neuropathic pain induced by Lumbar 5 ventral root transection in rat Pain 2006 123 306 321 10.1016/j.pain.2006.03.011 16675114

[B52] Wei F Guo W Zou S Ren K Dubner R Supraspinal glial-neuronal interactions contribute to descending pain facilitation J Neurosci 2008 28 10482 10495 10.1523/JNEUROSCI.3593-08.2008 18923025 PMC2660868

[B53] Horiuchi T Mitoma H Harashima S Tsukamoto H Shimoda T Transmembrane TNF-alpha: structure, function and interaction with anti-TNF agents Rheumatology (Oxford) 2010 49 1215 1228 10.1093/rheumatology/keq031 PMC2886310 20194223

[B54] DeLeo JA Tanga FY Tawfik VL Neuroimmune activation and neuroinflammation in chronic pain and opioid tolerance/hyperalgesia Neuroscientist 2004 10 40 52 10.1177/1073858403259950 14987447

[B55] Marchand F Perretti M McMahon SB Role of the immune system in chronic pain Nat Rev Neurosci 2005 6 521 532 15995723 10.1038/nrn1700

[B56] Watkins LR Maier SF Glia: a novel drug discovery target for clinical pain Nat Rev Drug Discov 2003 2 973 985 10.1038/nrd1251 14654796

[B57] Zimmermann M Ethical guidelines for investigations of experimental pain in conscious animals Pain 1983 16 109 110 10.1016/0304-3959(83)90201-4 6877845

[B58] Hao S Wolfe D Glorioso JC Mata M Fink DJ Effects of transgene-mediated endomorphin-2 in inflammatory pain Eur J Pain 2009 13 380 386 10.1016/j.ejpain.2008.05.008 18567517 PMC2656597

[B59] Chaplan SR Bach FW Pogrel JW Chung JM Yaksh TL Quantitative assessment of tactile allodynia in the rat paw J Neurosci Methods 1994 53 55 63 10.1016/0165-0270(94)90144-9 7990513

[B60] Dixon WJ Efficient analysis of experimental observations Annu Rev Pharmacol Toxicol 1980 20 441 462 10.1146/annurev.pa.20.040180.002301 7387124

[B61] Livak KJ Schmittgen TD Analysis of relative gene expression data using real-time quantitative PCR and the 2(-Delta Delta C(T)) Method Methods 2001 25 402 408 10.1006/meth.2001.1262 11846609

[B62] Hao S Liu S Zheng X Zheng W Ouyang H Mata M Fink DJ The role of TNFalpha in the periaqueductal gray during naloxone-precipitated morphine withdrawal in rats Neuropsychopharmacology 2011 36 664 676 10.1038/npp.2010.197 21068718 PMC3055683

[B63] Tan PH Yang LC Shih HC Lan KC Cheng JT Gene knockdown with intrathecal siRNA of NMDA receptor NR2B subunit reduces formalin-induced nociception in the rat Gene Ther 2005 12 59 66 10.1038/sj.gt.3302376 15470478

